# Evidence for the critical role of the PI3K signaling pathway in particulate matter-induced dysregulation of the inflammatory mediators COX-2/PGE_2_ and the associated epithelial barrier protein Filaggrin in the bronchial epithelium

**DOI:** 10.1007/s10565-019-09508-1

**Published:** 2019-12-28

**Authors:** Chenjian Song, Lingjing Liu, Junjie Chen, Yiran Hu, Jingli Li, Beibei Wang, Saverio Bellusci, Chengshui Chen, Nian Dong

**Affiliations:** 1 Department of Respiratory and Critical Care Medicine, The First Affiliated Hospital of Wenzhou Medical University, Wenzhou, China; 2 Cardio-Pulmonary Institute, Justus Liebig University, Giessen, Germany

**Keywords:** PM, PI3K/AKT, COX-2/PGE_2_, Bronchial epithelial damage, Filaggrin

## Abstract

Particulate matter (PM) is an environmental pollutant closely associated with human airway inflammation. However, the molecular mechanisms of PM-related airway inflammation remains to be fully elucidated. It is known that COX-2/PGE_2_ play key roles in the pathogenesis of airway inflammation. Filaggrin is a transmembrane protein contributing to tight junction barrier function. As such, Filaggrin prevents leakage of transported solutes and is therefore necessary for the maintenance of epithelial integrity. The objective of the present study was to investigate the regulatory mechanisms of COX-2/PGE_2_ and Filaggrin upon PM exposure both in vivo and in vitro. C57BL/6 mice received intratracheal instillation of PM for two consecutive days. In parallel, human bronchial epithelial cells (HBECs) were exposed to PM for 24 h. PM exposure resulted in airway inflammation together with upregulation of COX-2/PGE_2_ and downregulation of Filaggrin in mouse lungs. Corresponding dysregulation of COX-2/PGE_2_ and Filaggrin was also observed in HBECs subjected to PM. PM exposure led to the phosphorylation of ERK, JNK, and PI3K signaling pathways in a time-dependent manner, while blockade of PI3K with the specific molecular inhibitor LY294002 partially reversed the dysregulation of COX-2/PGE_2_ and Filaggrin. Moreover, pretreatment of HBECs with NS398, a specific molecular inhibitor of COX-2, and AH6809, a downstream PGE_2_ receptor inhibitor, reversed the downregulation of Filaggrin upon PM exposure. Taken together, these data demonstrated that the PI3K signaling pathway upregulated COX-2 as well as PGE_2_ and acted as a pivotal mediator in the downregulation of Filaggrin.

**Electronic supplementary material:**

The online version of this article (10.1007/s10565-019-09508-1) contains supplementary material, which is available to authorized users.

## Introduction

As industrialization has progressed worldwide, atmospheric pollution is recognized to be the leading contributor to global disease burden (Huang 2014). The major atmospheric pollutant, particulate matter (PM), is a mixture of solid particles and liquid droplets, which primarily deposits in the lungs through inhalation (Mukherjee and Agrawal 2018; Traboulsi et al. 2017). Substantial epidemiological investigations have revealed that PM exposure participates in the pathogenesis of airway diseases (Li et al. 2018b; Wang et al. 2018). Human bronchial epithelial cells (HBECs) act as the first line of defense against PM exposure, which ultimately leads to the malfunction of the cells (Chen et al. 2016; De Grove et al. 2018). The exposed HBECs secrete excessive cytokines, which in turn lead to irreversible pathological changes in HBECs (Wang et al. 2007). Considering the pivotal role of HBECs in the pathogenesis of airway diseases, research into the relevant mechanism affecting these cells might contribute to an understanding of PM-related airway disease.

Cyclooxygenase2 (COX-2) is an inducible rate-limiting enzyme, the expression of which is upregulated by mitogen and endotoxin (Rumzhum and Ammit 2016). COX-2 is a well-known inflammatory mediator, which mainly converts arachidonic acid into prostaglandins including prostaglandin E2 (PGE_2_) (Yagami et al. 2016). Previous research studies have demonstrated that PM exposure upregulates the expression of COX-2 in multiple systems including cardiovascular and neuron systems, and upregulated COX-2 subsequently exerts a variety of pathogenic effects. Jie et al. reported that COX-2 mediates PM-induced apoptosis of vascular endothelial cells in cardiovascular injury (Yin et al. 2017). Additionally, Ben et al. reported that PM exposure stimulated COX-2-mediated excitatory synaptic transmission of neurons in neurodegenerative disease (Li et al. 2018a). However, the pathogenic role of COX-2 in the malfunction of HBECs remains to be elucidated. Filaggrin belongs to the keratin gene family and is involved in the differentiation of epithelial cells (Zhong et al. 2005). The loss-of-function in mutations of *FLG* in humans is correlated with airway hyperresponsiveness, a characteristic of PM-related airway disease (Berg et al. 2012; Cubero et al. 2016; Palmer et al. 2007). A systemic review of the literature concerning COX-2 and Filaggrin pointed out that PM downregulates Filaggrin via COX-2 expression/PGE_2_ production, leading to skin barrier dysfunction (Lee et al. 2016). Given the functional similarity between the bronchial epithelium and the epidermis in maintaining epithelial barrier integrity, it is meaningful to investigate the expression of COX-2 and Filaggrin upon PM exposure and explore the potential relevant molecular mechanisms.

Therefore, an in vivo mouse model of PM exposure was adopted to investigate the corresponding lung morphological changes in addition to the expression pattern of COX-2 and Filaggrin. Moreover, a HBEC in vitro model of PM exposure was employed to explore the potential relevant mechanism. We report that PM exposure upregulates COX-2 and downregulates Filaggrin via the PI3K/AKT signaling pathway. We propose that COX-2, as well as downstream PGE_2_, leads to Filaggrin downregulation.

## Materials and methods

### Reagents and antibodies

The standard reference airborne PM (standard reference material 1649b), mainly composed of polycyclic aromatic hydrocarbons, polychlorinated biphenyl congeners, pesticides, and doxins, was purchased from the National Institute of Standards and Technology (Gaithersburg, MD, USA). The specific molecular inhibitors SP600125, U0126, LY294002, AH6809, and NS398 were purchased from Selleck (Houston, TX, USA). Antibodies against COX-2, phospho-ERK, ERK, phospho-JNK, JNK, phospho-AKT, AKT, and EpCAM were purchased from Cell Signaling Technology (Danvers, MA, USA). Anti-Filaggrin antibody was obtained from GeneTex (San Antonio, TX, USA) and anti-GAPDH antibody was purchased from Beyotime (Shanghai, China). mRNA primers were synthesized by Sangon Biotech (Shanghai, China). The reagents for real-time qPCR were purchased from TaKaRa Bio (Shiga, Japan). The reagents used for western blotting were obtained from Solarbio Life Science (Beijing, China).

### Cells and animals

HBECs were purchased from the Chinese Academy of Sciences (Shanghai, China) and cultured at 37 °C in a 5% CO_2_ incubator in RPMI-1640 medium (Gibco, Waltham, MA, USA) supplemented with 10% fetal bovine serum (Gibco, Waltham, MA, USA) and 50 U/mL penicillin and streptomycin (Gibco, Waltham, MA, USA). Male C57BL/6 mice (specific pathogen-free), with body weights ranging from 20 to 22 g, were purchased from Beijing Vital River Laboratory Animal Technology Company (Beijing, China). All protocols for animal experiments were approved by the Animal Experiment Center Ethics Committee, Wenzhou Medical University.

### In vitro and in vivo PM treatments

For in vitro experiments, PM was resuspended in PBS at a stock concentration of 4 mg/cm^3^ and HBECs were exposed to PM at 0, 25, 50, 100, 200, and 400 μg/cm^3^ for 24 h. Additionally, HBECs were pretreated with a specific molecular inhibitor 60 min prior to PM exposure. For in vivo experiments, PM was resuspended in PBS at 100 μg (in 25 μL PBS) and mice were exposed for two consecutive days via daily intratracheal instillation. All mice were sacrificed 24 h after the last intratracheal instillation of PM.

### Real-time qPCR

Total RNA was isolated through a guanidinium isothiocyanate/chloroform-based technique (TRIzol™ Invitrogen, Carlsbad, USA). RNA was subsequently reverse transcribed to cDNA using a SuperScript First-strand Synthesis System (Invitrogen, USA). SYBR Green PCR master kit was used with appropriate concentrations (10 nM) of forward and reverse primers in a total volume of 20 μL. Optimization was conducted for each gene-specific primer prior to the experiment to confirm that 10 nmol/L primer concentrations did not produce nonspecific primer-dimer amplification signals in no-template control wells. Quantitative RT-PCR was performed using an ABI 7000 PCR instrument (Eppendorf, Hamburg, Germany) with the two-stage program parameters provided by the manufacturer, as follows: 1 min at 95 °C and then 40 cycles of 5 s at 95 °C and 30 s at 60 °C. Sequences of the primer sets used for this analysis are as follows: COX-2: 5′-TGAGTGTGGGATTTGACCAG-3′ (forward [F]) and 5′-TGTGTTTGGAGTGGGTTTCA-3′ (reverse [R]); FLG: 5′-TGATGCAGTCTCCCTCTGTG-3′ (F) and 5′-TGTTTCTCTTGGGCTCTTGG-3′ (R); and for human glyceraldehyde-3-phosphate dehydrogenase (GAPDH): 5′-CCACCCATGGCAAATTCCATGGCA-3′ (F) and 5′-TCTACACGGCAGGTCAGGTCCACC-3′ (R). Specificity of the produced amplification product was confirmed by examination of dissociation reaction plots. Each sample was tested in triplicate with quantitative RT-PCR. Each group had six wells (Table 1).

**Table 1:** The sequence of primers

Genes	Forward	Reverse
*GAPDH*	5′-CCACCCATGGCAAATTCCATGGCA-3′	5′-TCTACACGGCAGGTCAGGTCCACC-3′
*COX2*	5′-TGAGTGTGGGATTTGACCAG-3′	5′-TGTGTTTGGAGTGGGTTTCA-3′
*FLG*	5′-TGATGCAGTCTCCCTCTGTG-3′	5′-TGTTTCTCTTGGGCTCTTGG-3′

### Western blotting

Total protein from lung tissues and HBECs were lysed in RIPA buffer containing phenylmethanesulfonyl fluoride (PMSF, Beyotime) and phosphatase inhibitor (Beyotime). The concentration was measured using a bicinchoninic acid (BCA) protein assay kit (Thermo Scientific, Waltham, MA, USA). An equal quantity of lysate (45 μg) was loaded onto a 10% sodium dodecyl sulfate polyacrylamide gel electrophoresis (SDS-PAGE) gel for electrophoresis after which the proteins were transferred to a PVDF membrane. The PVDF membrane was first incubated overnight at 4 °C with different dilutions of a primary antibody and then incubated with an appropriate secondary antibody for 1 h at room temperature. Immunoreactive bands were detected with a Bio-Rad Laboratories system using ECL reagents (Thermo Scientific, Waltham, MA, USA) and the relevant signals measured using Image Lab software.

### Histological analysis

The lungs were embedded in paraffin and 5-μm sections were generated and stained with hematoxylin and eosin (H&E) according to the manufacturer’s instructions. Histological analysis was based on a method previously described (Lee et al. 2006) using an Olympus BX53 inverted microscope (Olympus, Melville, NY, USA). Three independent blinded investigators graded the score. The degree of peribronchial and perivascular inflammation was evaluated on a subjective scale of 1 to 4. A value of 1 was adjudged when no inflammation was observed, a value of 2 for occasional cuffing with inflammatory cells, a value of 3 when most bronchi or vessels were surrounded by a thin layer (1 to 5) of inflammatory cells, and a value of 4 when most bronchi or vessels were surrounded by a thick layer (> 5) of inflammatory cells.

### Immunofluorescence staining

Mice exposed to PM for two consecutive days were sacrificed (24 h after the second PM treatment) and the lungs fixed in 4% paraformaldehyde, infiltrated with 30% sucrose/PBS under a pressure of 20 cmH_2_O, embedded in Tissue-Tek OCT compound (Sakura Finetek, Torrance, CA, USA), and then fresh-frozen in liquid nitrogen. Five- and 50-μm-thick sections were stored at − 80 °C and then fixed in ice-acetone for 10 min. The lung tissues were then incubated overnight with mouse monoclonal antibodies against EpCAM, COX-2, or Filaggrin, respectively, and then incubated with the corresponding secondary antibodies. Counterstaining was performed with DAPI (4,6′-diamidino-2-phenylindole). Confocal microscopic images were collected using a Leica TCS SL laser scanning confocal microscope (Leica Microsystems, Mannheim, Germany).

### Statistical analysis

All numerical results were presented as mean ± SD. At least three independent biological repeats of experiments were carried out for statistical analyses. A one-way ANOVA with Bonferroni post hoc test was used to evaluate statistical significance between data sets. The level of statistical significance was set at *P* < 0.05.

## Results

### In vivo PM exposure led to lung inflammation mainly confined to the conducting airway in C57BL/6 mice

**Fig. 1:**
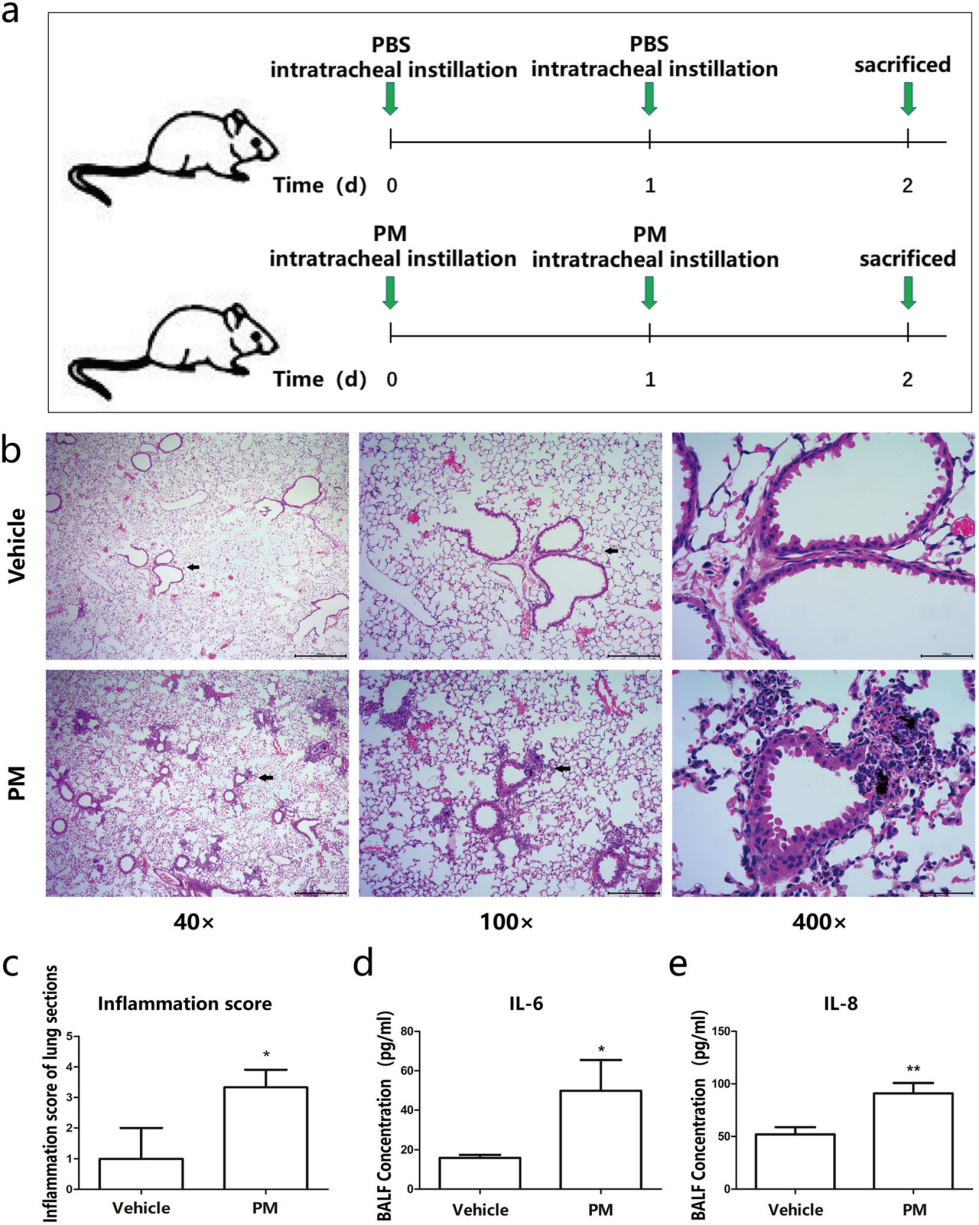
PM exposure in C57BL/6 mice leads to lung inflammation mainly confined to the conducting airways. **a** Protocol of PM exposure. **b** Representative images of lung sections stained with H&E. **c** Semiquantitative inflammatory score of lung sections stained with H&E. **d** Level of IL-6 in BALF measured by ELISA. **e** Level of IL-8 in BALF measured by ELISA. Values represent means ± SD, *P < 0.05 or **P < 0.01, compared with the vehicle group; *n* = 3

A mouse model of PM exposure was adopted with intratracheal instillation of 100 μg/day/mouse of PM suspension for two consecutive days (Fig. 1a). Vehicle (PBS only)-treated mice were used as controls (*n* = 8). Lung histological changes were evaluated through H&E staining between vehicle and PM-treated experimental mice (*n* = 8) (Fig. 1b). Conducting airway inflammation was observed in PM-treated mice. Supporting these histological changes, the proinflammatory cytokines IL-6 and IL-8 measured by ELISA were higher in the PM group compared to those in the vehicle group (Fig. 1d, e). These results indicate that PM exposure induces conducting airway inflammation.

### Dysregulation of COX-2/PGE_2_ and Filaggrin in mouse lungs upon PM exposure in vivo

**Fig. 2:**
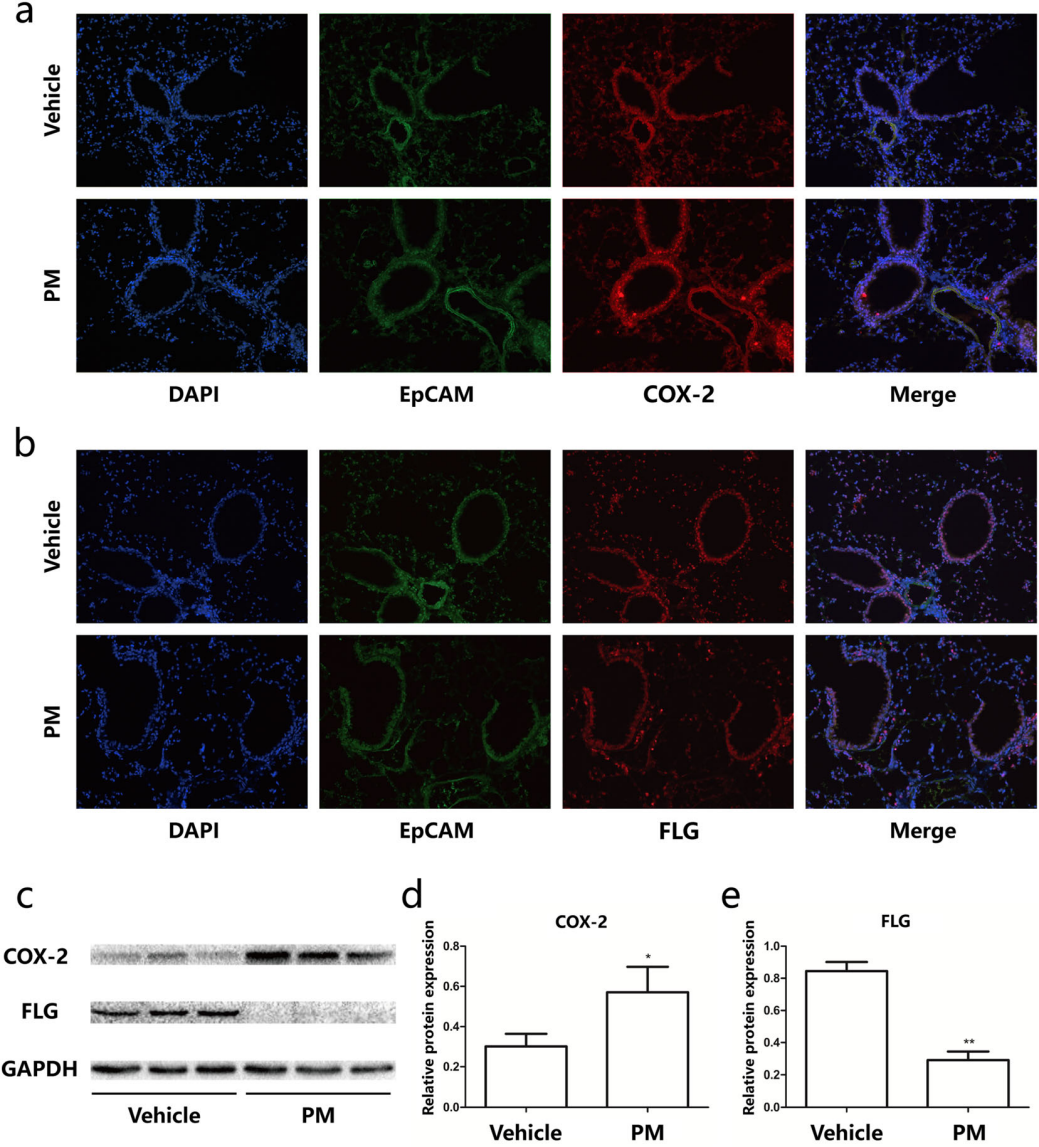
Dysregulation of COX-2/PGE_2_ and Filaggrin in mouse lungs upon PM exposure. Immunofluorescence staining of lung sections from mice challenged with PM for two consecutive days: EpCAM (green), COX-2/Filaggrin (red), and DAPI (blue), respectively. **a** Representative image of COX-2 (red) in lung tissues. **b** Representative image of Filaggrin (red) in lung tissues. **c** Western blot analysis of the expression of COX-2 and Filaggrin in whole lung tissue. The optical densities of protein bands are illustrated in **d** and **e**. Values represent means ± SD, *P < 0.05 or **P < 0.01, compared with the vehicle group; *n* = 3

Next, we explored the potential dysregulation of COX-2/PGE_2_ and Filaggrin in lung tissues of mice upon PM exposure. Immunohistochemical staining revealed slightly elevated COX-2 expression in the bronchial epithelium of lung tissues (Fig. 2a). In addition, immunohistochemical staining showed slightly downregulated Filaggrin around the bronchial epithelium (Fig. 2b). This result was also confirmed by western blot analysis (Fig. 2c–e). In summary, PM exposure of the conducting airway of the mice led to the dysregulation of COX-2 and Filaggrin.

### PM exposure induced the dysregulation of COX-2/PGE_2_ and Filaggrin in HBECs in vitro

**Fig. 3:**
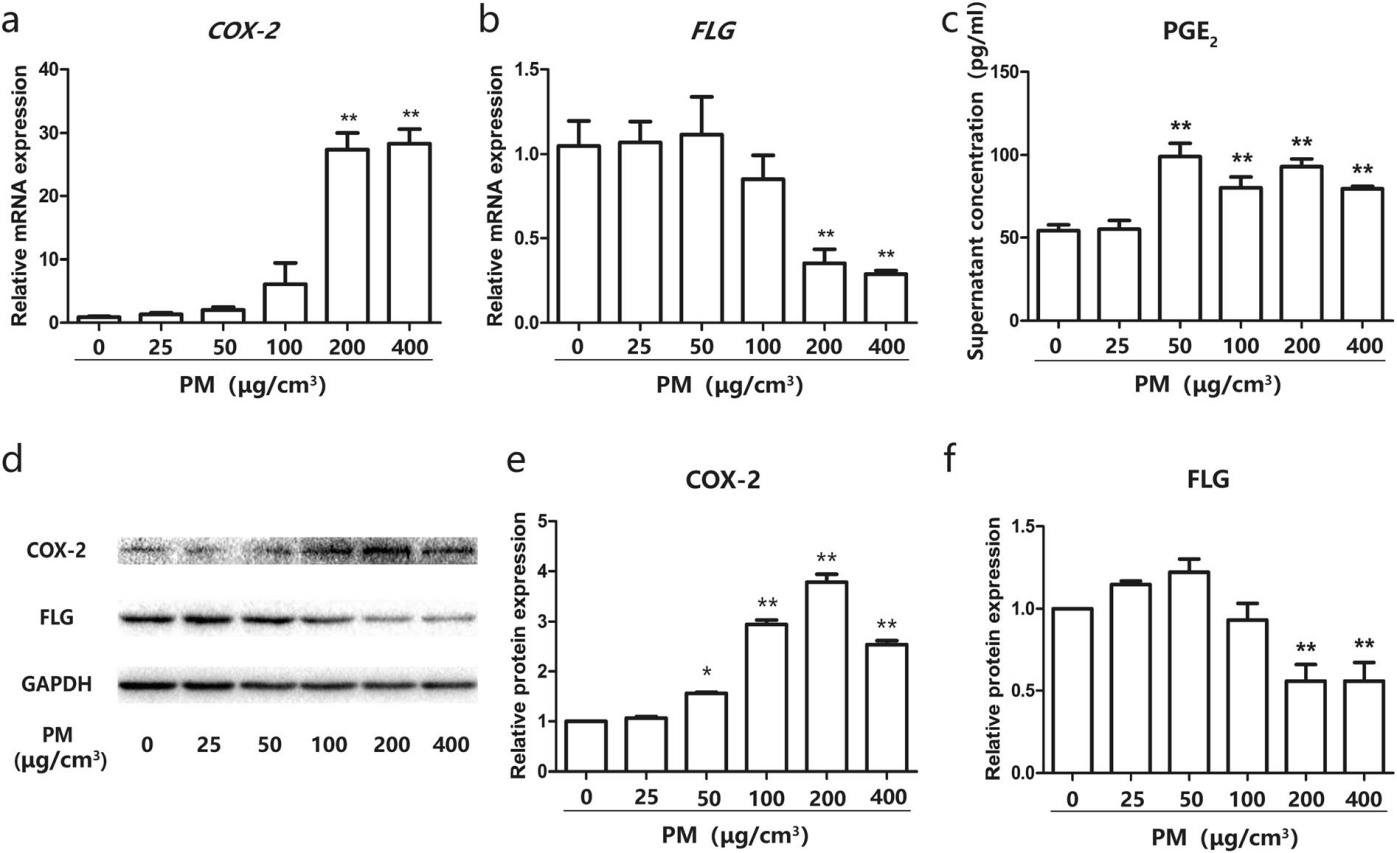
PM exposure induced the dysregulation of COX-2/PGE_2_ and Filaggrin in HBECs. **a**, **b** HBECs were challenged with PM in a dose-dependent (0, 25, 50, 100, 200, and 400 μg/cm^3^) for 24 h. The mRNA of COX-2 and Filaggrin were measured by real-time PCR. **c** The level of PGE_2_ in supernatant was measured by ELISA. **d** The protein expression of COX-2 and Filaggrin were measured by western blot analysis. The optical densities of protein bands are illustrated in **e** and **f**. Values represent means ± SD, *P < 0.05 or **P < 0.01, compared with the vehicle group; *n* = 3

To validate the dysregulation of COX-2/PGE_2_ and Filaggrin in vitro, human bronchial epithelial cells (HBECs, also termed BEAS-2B, an epithelial virus-transformed cell line) were stimulated with increasing doses of PM. Our results indicate that PM induced *COX-2* mRNA and protein upregulation in HBECs in a dose-dependent manner. Upregulation of PGE_2_ at the protein level was also observed (Fig. 3a, c, d, e). Conversely, PM induced the downregulation of *Filaggrin* mRNA and protein in HBECs in a dose-dependent manner (Fig. 3b, d, f). These data demonstrated that the effect of in vivo PM exposure on COX-2/PGE_2_ and Filaggrin expression can be reproduced in vitro using HBECs, thereby opening the way to more mechanistic insights into this regulation. Some of these mechanisms are explored in the following set of experiments.

### PM induced the dysregulation of COX-2/PGE_2_ and Filaggrin via activation of the PI3K pathway

**Fig. 4:**
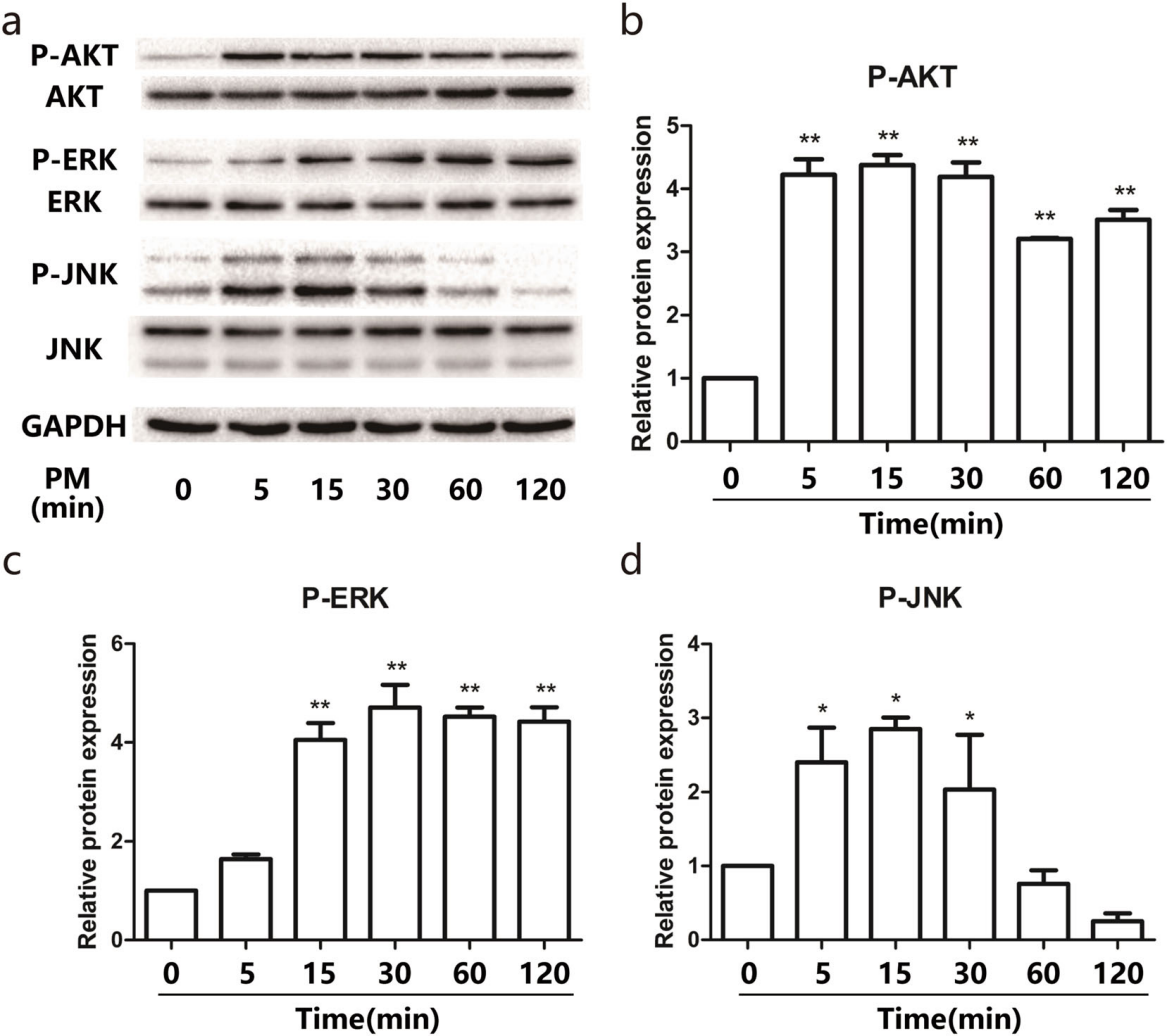
PM induced phosphorylation of MAPK and PI3K pathways in a time-dependent manner. **a** HBECs were stimulated with 200 μg/cm^3^ PM in a time-dependent manner (0, 5, 15, 30, 60, and 120 min). The phosphorylation of ERK, JNK, and PI3K were measured by western blotting. The optical densities of p-AKT/AKT, p-ERK/ERK, and p-JNK/JNK bands are illustrated in **b**, **c**, and **d**. Values represent means ± SD, *P < 0.05 or **P < 0.01, compared with the vehicle group; #*P* < 0.05 or ##*P* < 0.01, compared with the PM group; *n* = 3

**Fig. 5:**
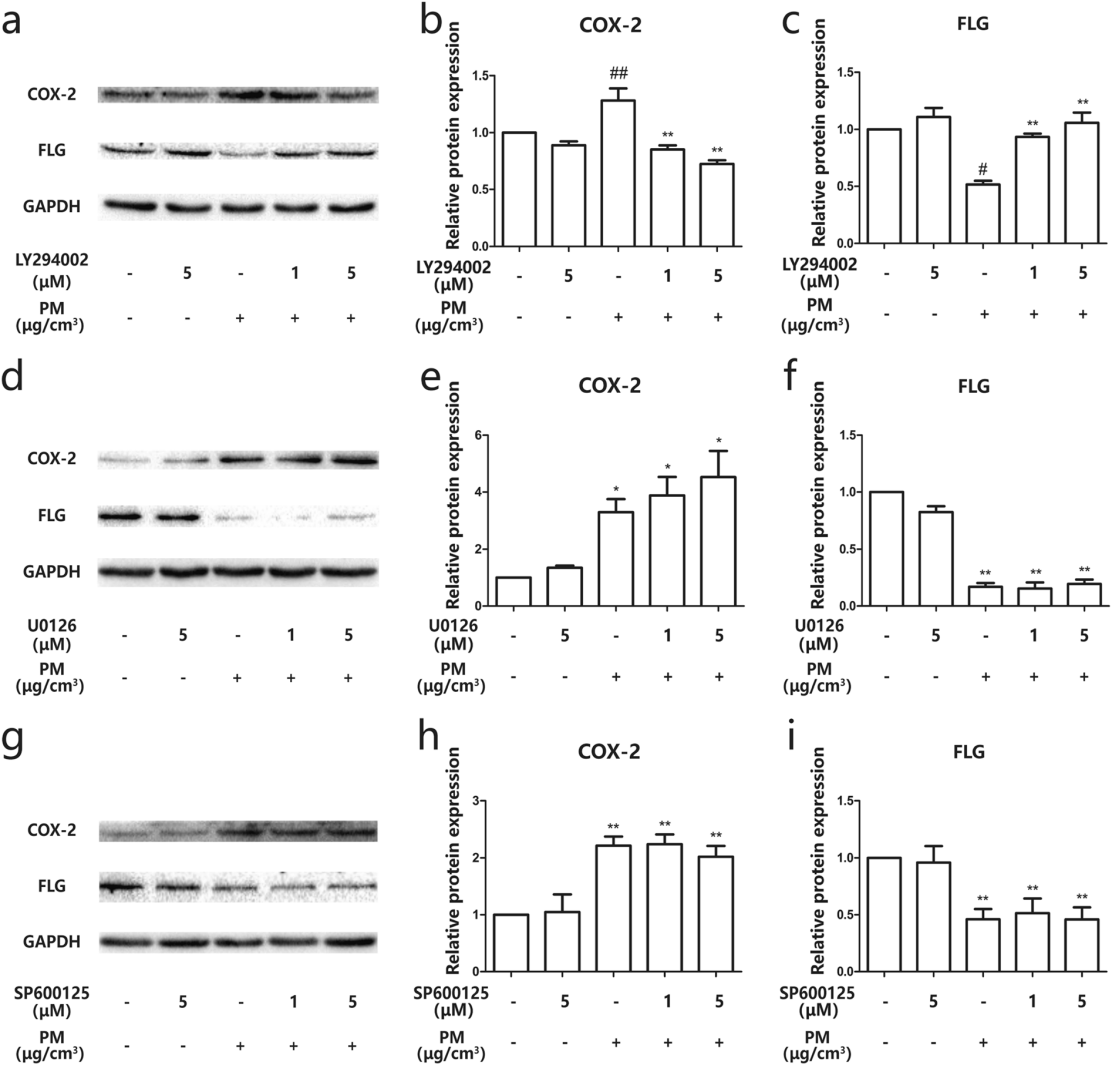
PM induced the dysregulation of COX-2/PGE_2_ and Filaggrin via the PI3K pathway. **a**, **d**, **g** HBECs were pretreated with specific molecular inhibitors of PI3K, ERK, and JNK and then stimulated with PM. The protein expression of COX-2 and Filaggrin were measured by western blot analysis. The optical densities of protein bands are illustrated in **b**, **c**, **e**, **f**, **h**, and **i**. Values represent means ± SD, *P < 0.05 or **P < 0.01, compared with the vehicle group; #*P* < 0.05 or ##*P* < 0.01, compared with the PM group; *n* = 3

To investigate the signaling pathway involved in the process of PM exposure, HEBCs were stimulated with PM for different times. The results of western blot analysis demonstrated that PM led to activation of ERK, JNK, and PI3K signaling in a time-dependent manner, respectively. The phosphorylation level of p-ERK and p-JNK reached at a peak 15 min after exposure to PM, while p-AKT reached a peak after 5 min (Fig. 4a–d). Pretreatment of HBECs with a specific PI3K inhibitor 1 h prior to PM exposure blocked the PM-induced dysregulation of COX-2/PGE_2_ and Filaggrin in a dose-dependent manner (Fig. 5a–c), while specific ERK and JNK inhibitors did not work (Fig. 5d–j). These data illustrated that PM exposure triggered changes in COX-2/PGE_2_ and Filaggrin expression via activation of the PI3K signaling pathway.

### Targeting COX-2/PGE2 partially reversed PM-induced downregulation of Filaggrin

**Fig. 6:**
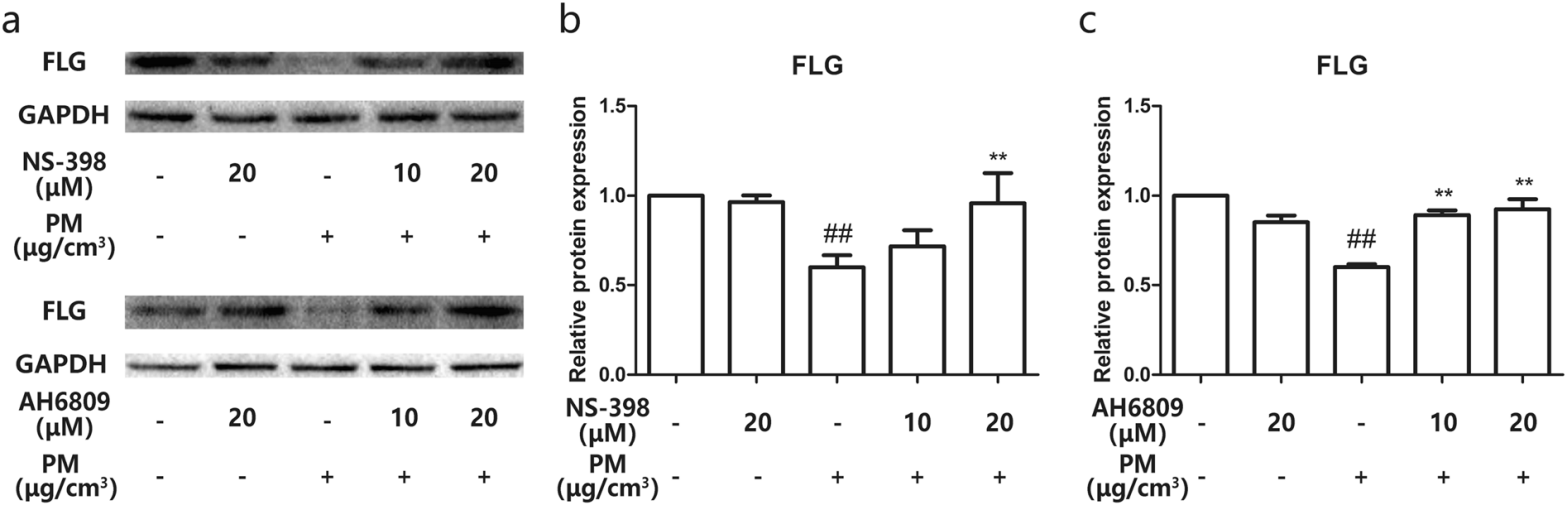
Targeting COX-2/PGE_2_ partially reversed PM-induced downregulation of Filaggrin. **a** HBECs were pretreated with specific molecular inhibitors of COX-2 and PGE2 receptor then stimulated with PM. The protein expression of Filaggrin was measured by western blotting. The optical densities of protein bands are illustrated in **b** and **c**. Values represent means ± SD, *P < 0.05 or **P < 0.01, compared with the vehicle group; #*P* < 0.05 or ##*P* < 0.01, compared with the PM group; *n* = 3

To elucidate the potential effect of COX-2/PGE_2_ in the downregulation of Filaggrin expression upon PM exposure, HBECs were pretreated with specific COX-2/PGE_2_ receptor inhibitors before PM exposure. Both COX-2 inhibitor  and PGE_2_ receptor inhibitor prevented PM-induced downregulation of Filaggrin in a dose-dependent manner (Fig. 6a–c). Our results, therefore, indicate that downregulation of Filaggrin upon PM exposure is mediated via the upregulation of COX-2/PGE_2_.

## Discussion

A growing body of epidemiological evidence suggests that exposure to PM is closely correlated with the mortality and morbidity of respiratory diseases (Kim et al. 2017). However, the pathophysiological processes triggered by PM exposure are complex, involving mucus hyperproduction, bronchial epithelial injury, and impaired airway antibacterial defense leading to loss of epithelial integrity (Aloui et al. 2016; Chen et al. 2018; Xu et al. 2018a; b). COX-2 is a well-known critical mediator of the inflammatory process responsible for the synthesis of downstream PGE_2_. Numerous researchers have previously reported ROS-dependent upregulation of COX-2 in HBECs upon PM exposure (Fernando et al. 2019; Wang et al. 2017), while the effect of COX-2 in the differentiation of epithelial cells was scarcely analyzed. In comparison with the diverse effects of COX-2, the bronchial epithelial expression of Filaggrin is scarcely reported. For example, Ying et al. (2006) demonstrated a lack of Filaggrin expression in normal bronchial mucosa, while GeneCards (http://www.genecards.org) indicated that Filaggrin was expressed in the bronchial epithelium. The published literature focusing on Filaggrin is mainly restricted to a skin condition known as dermatosis (Batista et al. 2015; Orfali et al. 2017; Proksch et al. 2016; Scharschmidt et al. 2009). Loss of epidermal Filaggrin expression correlates with allergic sensitization through enhanced percutaneous exposure to environmental substances, which, in turn, provokes an adaptive immune response (Jones 2007; Saunders et al. 2016). As hyperresponsiveness is a characteristic of PM-related airway disease (Yang et al. 2019), whether this is mediated via decreased bronchial epithelial Filaggrin expression remains yet to be clarified.

Our work indicated that short-term PM exposure primarily leads to bronchitis, not alveolitis. Considering the difficulty in primary culture of bronchial epithelial cells from the mouse, immortalized HBECs were adopted for in vitro experiments, which might be of greater translational potential. The present research further indicated that COX-2 is primarily localized in the bronchial epithelium. Associated with such expression, we have reported for the first time Filaggrin downregulation in the bronchial epithelium upon PM exposure. Given the pivotal role of Filaggrin in the differentiation of epithelial cells, downregulated Filaggrin expression might lead to impaired epithelial integrity. As such, Filaggrin is a promising therapeutic target and future research into the function of Filaggrin is required. Using loss of function approaches in vitro for FLG will allow a determination of whether a decrease in FLG is sufficient to mimic the effect of PM exposure in the bronchial epithelium. In addition, the maintenance of an epithelial barrier using air–liquid interface in vitro culture systems could be established. Furthermore, in vivo experiments allowing *FLG* overexpression in the bronchial epithelium in the context of PM exposure (corresponding to a rescue experiment) will allow us to establish whether decreased FLG in pathological circumstances is required to elicit a deleterious epithelial phenotype in addition to impacting inflammation. Finally, it will be important to validate the results obtained so far in vitro and in vivo with PM in humans. Of particular interest will be the analysis of the BALF from humans exposed to different levels of PMs. Such populations are unfortunately not available in China. A quite recent development allowing gaps between basic and translational research to be filled has been the use of human lungs either from end-stage patients or from patients who died from nonrespiratory diseases. Precision cut lung slides allow the testing in vitro of the response of human lungs to different stimuli. The readouts include analysis by immunofluorescence, qPCR, and flow cytometry for different specific cell populations in addition to isolation of well-defined epithelial stem cells by FACS and evaluation of their self-renewal and differentiation capabilities using organoid assays (Gkatzis et al. 2018).

It has been previously reported that oxidative stress is primordial to explain the toxic effects generated by PM exposure. Important studies have demonstrated that PM exposure leads to ROS-dependent activation of signaling pathways mediating the pathological processes, including the MAPK signaling pathway (Dou et al. 2018; Kim et al. 2019; Liu et al. 2019; Tsai et al. 2017). Previous research reported ERK, JNK, and PI3K/AKT activation in the lung following lipopolysaccharide stimulation (Chen et al. 2011; Shi et al. 2018). In our PM exposure model, we also demonstrated the corresponding phosphorylation of ERK, JNK, and PI3K/AKT. The specific molecular inhibitors of ERK, JNK, and PI3K were used to investigate the role of these signaling pathways in PM-exposed HBECs. We demonstrated that the PI3K signaling pathway is dominant in mediating the dysregulation of COX-2 and Filaggrin, illustrated by the results of upregulation of COX-2/PGE_2_ and downregulation of Filaggrin being reversed by a specific molecular inhibitor of PI3K. The PM/PI3K/AKT axis appears to be sufficient to explain the upregulation of COX-2/PGE_2_ and downregulation of Filaggrin. In addition, COX-2 and PGE_2_ are themselves key mediators of Filaggrin downregulation.

**Fig. 7:**
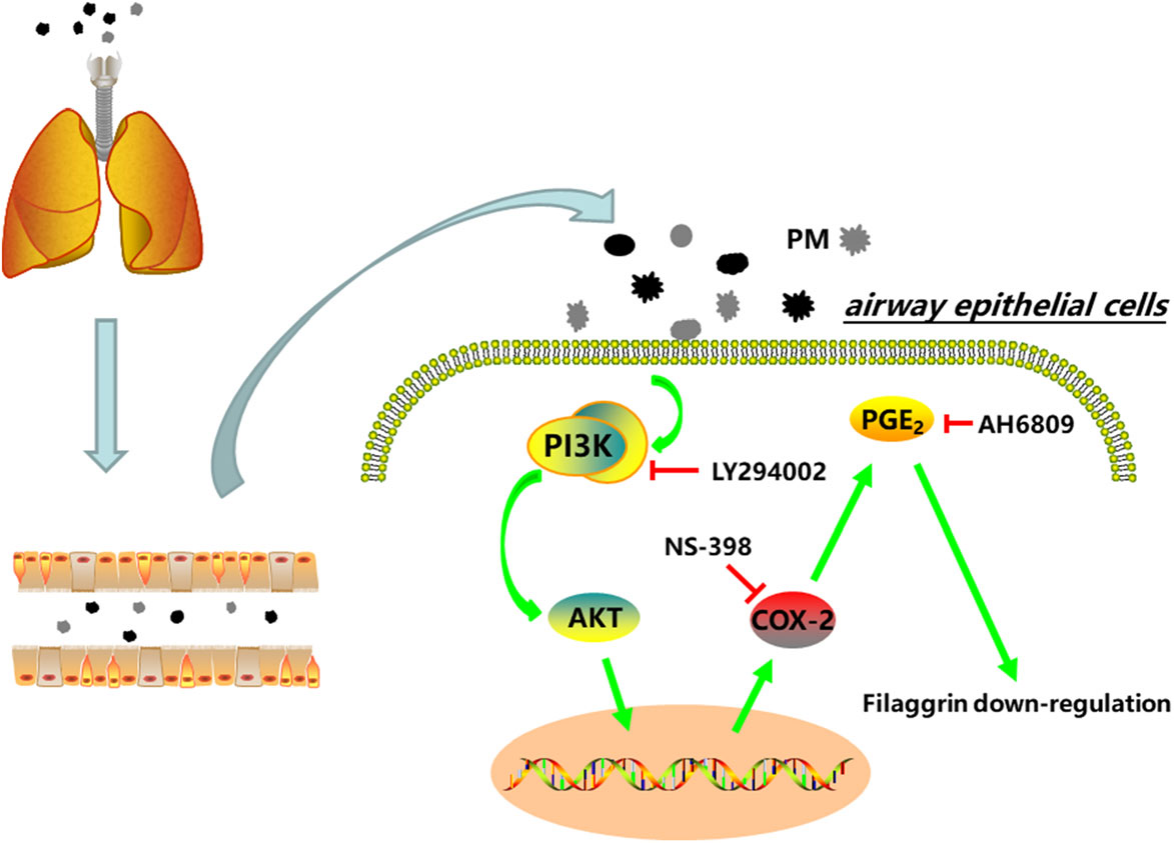
Mechanism of PM-induced airway epithelial injury (schematic). PM enters the airway by respiratory movement of the human body and deposits on the airway epithelial cells. PM activates the PI3K/AKT pathway in the airway epithelium and upregulates the expression of inflammatory factors such as COX-2, PGE_2_, IL-6, and IL-8, resulting in airway epithelial inflammation. Subsequently, airway epithelial inflammation causes a downregulation of Filaggrin expression via the COX-2/PGE_2_ pathway

Further research will be required to investigate the pathogenic role associated with *Filaggrin* gene dysregulation in PM-related airway disease not only under short-term conditions but also long-term PM exposure. Both in vivo and in vitro approaches will be useful to delineate the molecular mechanisms involved. For example, using the HBECs, it will be important to delineate the dynamic transcriptional changes occurring after PM exposure. This will likely allow the determination of the identity of important functional targets downstream of PMs. In addition, the cell line used for this study, HBECs, likely represents a heterogeneous cell population, which remains to be further defined. As the bronchial epithelium is composed of basal cells, club cells, variants of club cells, ciliated cells, goblet cells, and neuroendocrine cells, the identity of the cells which have been transformed and their proliferative and differentiation capabilities are unknown. Using single cell transcriptomics, the response to PM exposure of the different subpopulations present in HBECs could be determined. In particular, this will allow identification of the trajectory of differentiation (or de-differentiation if the cells affected are indeed already differentiated) in the context of PM exposure (Fig. 7).

In summary, the present research illustrated a critical role of the PI3K signaling pathway in PM-induced dysregulation of COX-2/PGE_2_ and Filaggrin, both in vitro and in vivo. Upregulated COX-2 and production of PGE_2_ leads to the downregulation of Filaggrin in the bronchial epithelium, which we propose is causative for the disruption of epithelial integrity. Future development of novel therapies targeting the PM/PI3K/AKT/COX-2/PGE_2_/Filaggrin axis is promising in protecting against PM-related destruction of epithelial integrity in humans.

## Electronic supplementary material

Figure S1 PM induced the dysregulation of PGE_2_ via the PI3K pathway. HBECs were pretreated with specific molecular inhibitors of ERK/JNK/PI3K then stimulated with PM. The protein expression of PGE_2_ was measured by ELISA. Values represent means ± SD, *: P < 0.05 or **: P < 0.01, compared with the Vehicle group; #: P < 0.05 or ##: P < 0.01, compared with the PM group; *n* = 3 (PNG 75 kb)

High resolution image (TIF 8723 kb)
