# Supplementary figures and images for: Evidence for the critical role of the PI3K signaling pathway in particulate matter-induced dysregulation of the inflammatory mediators COX-2/PGE2 and the associated epithelial barrier protein Filaggrin in the bronchial epithelium
Source: Cell Biol Toxicol. 2019 Dec 28;36(4):301–13. doi: 10.1007/s10565-019-09508-1 (PMC7363729; doi:10.1007/s10565-019-09508-1)

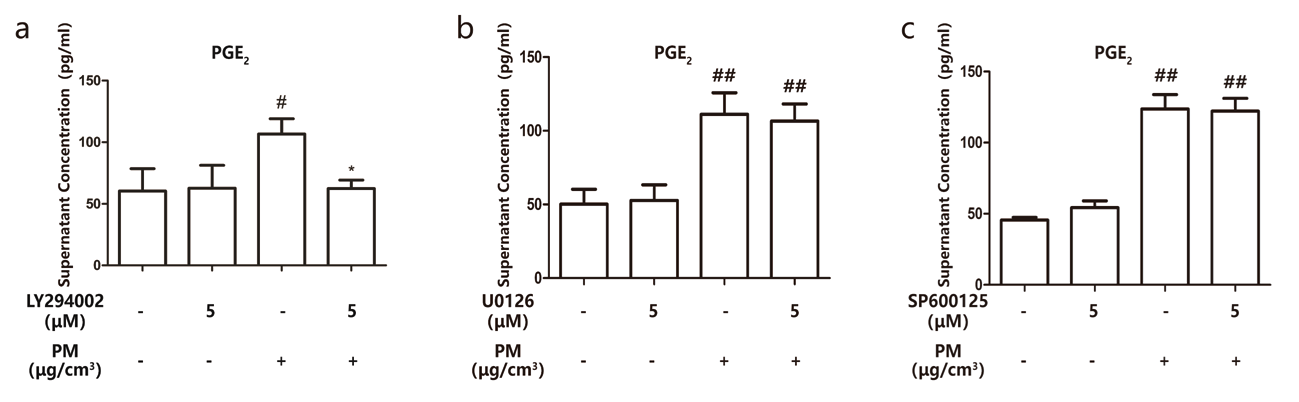

Supplement: Supplementary file 1 — PM induced the dysregulation of PGE2 via the PI3K pathway. HBECs were pretreated with specific molecular inhibitors of ERK/JNK/PI3K then stimulated with PM. The protein expression of PGE2 was measured by ELISA. Values represent means ± SD, *: P < 0.05 or **: P < 0.01, compared with the Vehicle group; #: P < 0.05 or ##: P < 0.01, compared with the PM group; n = 3 (PNG 75 kb) [file 10565_2019_9508_Fig8_ESM.png]

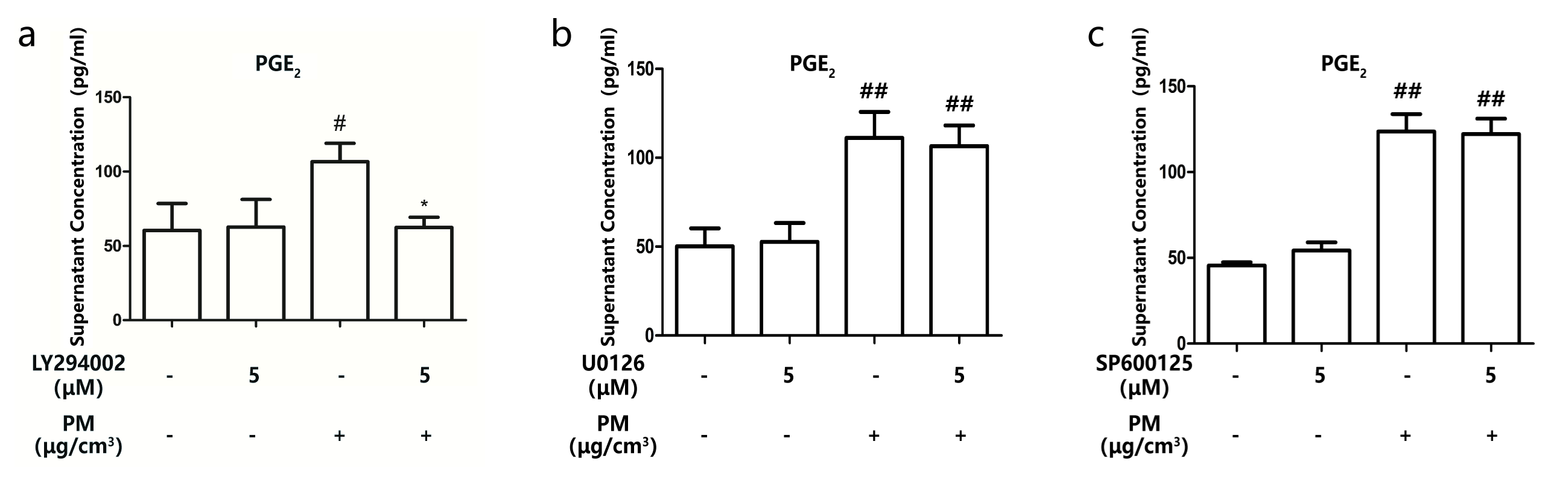

Supplement: Supplementary file 2 — High resolution image (TIF 8723 kb) [file 10565_2019_9508_MOESM1_ESM.tif]
